# From process to product: writing engagement and performance of EFL learners under computer-generated feedback instruction

**DOI:** 10.3389/fpsyg.2023.1258286

**Published:** 2023-10-23

**Authors:** Chen Shen, Penghai Shi, Jirong Guo, Suyun Xu, Jiwei Tian

**Affiliations:** ^1^Department of Foreign Languages, Xi’an Jiaotong University City College, Xi’an, China; ^2^School of Foreign Languages, Chang’an University, Xi’an, China; ^3^School of Foreign Studies, Xi’an Jiaotong University, Xi’an, China; ^4^ATC Navigation College, Air Force Engineering University, Xi’an, China

**Keywords:** automated writing evaluation, language proficiency, computer-assisted language learning, writing performance, CAF, engagement

## Abstract

Artificial intelligence (AI) technology is gradually penetrating the domain of education, opening up many possibilities for teaching and learning. Many educators, faced with the burden of commenting on substantial student essays, have introduced automated writing evaluation (AWE) into second language (L2) writing considering its affordance of immediate scores and diagnostic information. However, students’ processing strategies and perceptions of such computer-generated feedback and its impact on student writing quality, particularly as mediated by language proficiency, remain under-explored. This study examines the impact of *Pigai*, a Chinese AWE system, on revision processes and writing products of 42 English as a foreign language (EFL) learners with varying language levels by analyzing feedback points, feedback uptake, text quality in complexity, accuracy, and fluency (CAF), and perceptions. The findings are as follows. When confronted with AWE instruction, the majority of student work focuses on correcting errors, but higher-level students exhibit an emphasis on language improvement beyond the surface level compared to lower-level students. According to CAF measures, automated feedback exerts greater effects on accuracy for unskilled learners and lexical complexity for skilled learners in the development of interlanguage. Learner profiles and perceptions of students at different levels are explored along four dimensions: writing quality, cognitive engagement, behavioral engagement, and affective engagement. Finally, the potential issues of such technology-based writing instruction are pointed out.

## 1. Introduction

Feedback plays a pivotal role in L2 writing instruction by helping students realize the gap between what they already know and what they are expected to know with the aim of stimulating the ongoing progression of their target language (Reynolds et al., 2021; Zhai and Ma, 2022). In general, feedback comes from an agent such as a teacher, peer, or computer and possibly any combination of them (Wilson and Czik, 2016). Among these, computerized feedback, also known as automated writing evaluation (AWE) feedback, makes it possible for computer-assisted language learning (CALL) by leveraging cutting-edge natural language processing to instantly assess scores and the correctness/incorrectness of an essay. AWE is recognized as an application of artificial intelligence (AI) technology in language teaching and learning. The design has been purported to liberate teachers from the heavy workloads of reviewing writing (Zhang, 2020).

The rise of AWE programs has spawned a wealth of research. Previous studies primarily considered the feasibility of integrating new technologies into classroom instruction, thereby concentrating on verifying the consistency between computer-generated and manual scoring (Li et al., 2014). Gradually, scholarly concerns have been raised about AWE feedback’s possible positive or negative impacts on the primary stakeholders - students (Link et al., 2022). Most of them have taken a product-based approach to examine students’ short- or long-term writing quality guided by AWE feedback with coarse-grained metrics such as holistic score and/or accuracy (James, 2006; Choi, 2010; Kellogg et al., 2010; Li et al., 2015, 2017; Hassanzadeh and Fotoohnejad, 2021; Reynolds et al., 2021). However, the exclusive focus on holistic scores and/or accuracy is insufficient to account for the development of learners’ language levels. It is advisable to employ more comprehensive linguistic indices to capture the multi-dimensional changes in students’ writing with the mediation of AWE (Wilson and Czik, 2016). Because language is a complex, dynamic system, language acquisition cannot be fully explained by the performance of any one subsystem (Larsen-Freeman, 2006). Measures of complexity, accuracy, and fluency (CAF) will facilitate a more precise characterization of learners’ developmental dynamics of language and a more objective assessment of the effects of AWE on language progress (Wolfe-Quintero et al., 1998; Larsen-Freeman, 2006; Larsen-Freeman and Cameron, 2008). Research, notably on L2 writing, has demonstrated that written production can be featured by improvement or deterioration in one or more of the CAF components (Link et al., 2022). Thus, CAF will be employed in this study as significant writing competence indices to observe the effectiveness of AWE feedback on learners’ writing improvements.

Current research interest in automated assessment engines has remained in the validation of their psychometric properties (Chen and Cheng, 2008; Wilson and Czik, 2016), while the results are relatively inconsistent and insufficiently informative (Chapelle et al., 2015; Link et al., 2022). This study suggests that in order to provide clearer and more reasonable support for the conclusions drawn from assessing students’ writing outcomes, it is also important to take into account their revision process. By incorporating a process-based approach to investigate how students respond to and engage in AWE feedback, we can better understand the mediating factors that influence students’ promotion or inhibition of AWE use and writing quality. This will allow us to make more recommendations for future improvements in AWE technology and L2 writing pedagogy.

The inclusion of language proficiency to evaluate AWE instruments is necessary because research on EFL learners with varying proficiency levels can help gain more nuanced insights into the efficacy of AWE systems (Chen and Cheng, 2008; Xu and Zhang, 2022) but few studies have contained it as a variable. Learners of different language levels benefit differently from interactions with AWE, and thus the issue deserves further exploration. In summary, this study aims to explore the AWE-supported revision processes and written products of EFL students with different language proficiency based on a longitudinal, dynamic study with a mixed method.

## 2. Literature review

According to Warschauer and Ware (2006), research on the application of AWE systems in pedagogical contexts can be categorized into three areas: process, product, and process/product. Process-oriented research attempts to answer the question of how AWE software is used. Product-oriented research, on the other hand, primarily discusses whether AWE software can enhance writing outcomes. Research that follows a process/product-based approach is concerned with the interaction of use and outcome (Warschauer and Ware, 2006).

### 2.1. Product-based research on AWE

Most existing studies have adopted a product-based approach to examine the efficacy of a particular AWE system with an emphasis on L2 written production. Holistic score and writing accuracy are the most popular measures in the AWE literature, but the results remain inconclusive (Stevenson and Phakiti, 2014). Choi (2010) evaluated the effects of an AWE system called *Criterion* on holistic scores and accuracy of writing in two English learning settings (ESL and EFL). The research set up three integration levels: no-AWE, optional-AWE, and integrated-AWE. Results illustrated that the integrated-AWE group outperformed the no-AWE and optional-AWE groups in improving ESL and EFL learners’ writing quality. When measuring the pre/post-test and within-essay effects of integrated-AWE, holistic scores and accuracy increased significantly from the original to the revised drafts. However, those improvements did not appear in a comparison of the pre-test and post-test essays. Accordingly, AWE revealed immediate gains and limited transfer effects on the written output of L2 students.

Likewise, Kellogg et al. (2010) designed three conditions with no feedback, intermittent feedback, and continuous feedback to measure the changes in holistic scores and accuracy under *Criterion* instruction. Participants were asked to complete and revise three essays based on different feedback conditions and write a post-test essay without the AWE intervention to assess the transfer of learning. The study supports the partial conclusion of Choi (2010) that users who received continuous feedback were the most beneficial and did not show a significant increase in overall scores. Nevertheless, the transfer effects of error reduction, especially in mechanics, usage, grammar, and style, were found in this study, which contradicts Choi’s (2010) finding that there was no long-term improvement in writing accuracy.

Fan (2023) employed a quasi-experimental design to investigate the impact of *Grammarly* on the quality of EFL students’ writing. The experiment consisted of two rounds including a treatment group that received both automated and teacher feedback and a control group that merely accepted teacher feedback. From the posttest results, no significant differences were found between the treatment group and the control group on complexity, accuracy, and fluency measures. The study reported on the ineffectiveness of the AWE system for writing enhancement. The same measuring dimensions were adopted by Xu and Zhang (2022) to conduct a naturalistic classroom study targeting EFL students at different proficiency levels under the instruction of the AWE, *Pigai*. However, the results of the two studies apparently diverged. In their research, findings manifested no differences in lexical and syntactic complexity in the pretest and no significant variation in syntactic complexity and accuracy in the posttest. The invariant accuracy of high-proficiency students and the increased accuracy of low-proficiency students yielded a major effect of AWE on the writing accuracy of low-level learners.

The inconsistent conclusions might arise from the differences in the research subjects and length of experiments in the two studies. Xu and Zhang (2022) only selected four metrics for macroscopic observation of changes in CAF, and the performance of students with different levels of proficiency in concrete dimensions, such as coordination, subordination, and lexical richness, was not thoroughly analyzed. Additionally, both studies lacked qualitative approaches to analyze how students used AWE feedback in their revision process. This is the interface between the provision of feedback and the results generated by the feedback, which merits further exploration.

### 2.2. Process-based research on AWE

The key issue with any product-based research is that it treats the revision process as a black box (Warschauer and Ware, 2006). The advent of process-based research has made it possible to access the inside of this black box. Though few studies have paid attention to the revision process of EFL learners, their emphasis is different. Chapelle et al. (2015) incorporated multiple validity evidence into their research, including feedback accuracy, student operations, uptake, and perceptions of feedback, to argue for the validation of such formative assessment, which helped develop a more systematic framework for appraising AWE in relation to revision processes. Chodorow et al. (2010) evaluated the error correction capability of two AWE systems for articles and prepositions by collecting data on user actions in response to feedback. The findings showed that 13% of participants ignored suggested corrections, 33% browsed them without taking action, 22% noticed them and triggered online searches, and 32% included them in revisions. Bai and Hu (2017) identified the accuracy of automated feedback and students’ uptake rates based on different corrective categories. The results reported correct rates of 98, 58 and 22% for *Pigai*’s feedback in mechanical, grammatical and collocational aspects; their corresponding uptake rates were 74, 51 and 12%. The findings demonstrated that users’ responses to automated feedback were heavily reliant on its accuracy.

Most scholars assessed AWE systems around how students engaged in the revision process primarily by qualitative instruments, such as interviews, self-reports, verbal protocols, and observations (Koltovskaia, 2020; Lee, 2020; Zhang, 2020; Fu and Liu, 2022). They concur that engagement is a key mediating variable for this kind of formative feedback to influence learners’ writing development. Engagement is constructed as a multidimensional framework that generally involves cognitive, behavioral, and affective engagement. According to Koltovskaia’s (2020) definition based on the AWE context, cognitive engagement refers to the metacognitive and cognitive strategies that students employ to process AWE feedback. Behavioral engagement revolves around students’ revision operations and strategies as well as time allocations for AWE feedback. Affective engagement is concerned with students’ emotional and attitudinal reactions to AWE feedback. Learners’ varied engagement in these three dimensions may result in different responses to AWE feedback, which in turn affects their writing outcomes. In this process, individual factors (e.g., learning beliefs, language proficiency), as well as contextual factors (e.g., task load, teacher stance), play a mediating role in learners’ engagement (Zhang, 2020). This study follows the engagement framework combining quantitative and qualitative methods to explore the discrepancies in perceptions of technology-based feedback among students of different language levels.

### 2.3. Process or product-based research on AWE

To date, a dearth of research has elucidated the association between L2 learners’ revision processes and products generated by AWE. Liao (2016) assigned four writing tasks to 63 EFL learners who received guidance from *Criterion* to investigate their grammatical accuracy development, perceptions, and possible factors mediating acquisition. Quantitative analysis showed that students’ errors in new texts were not significantly reduced until the first draft of the third paper. Qualitative data identified the mediating role of agency, repetitive behaviors, and metacognitive strategies in the revision process to facilitate correction completion.

Link et al. (2022) conducted a control experiment to determine whether AWE tools should be recommended as a complement to teacher feedback. Participants in the experimental group received linguistic feedback from AWE and content feedback from a teacher, and the control group received all feedback from the teacher. The normalized frequencies of feedback provided from different agencies and the revision operations taken by students were calculated to track the effect of AWE and instructors on the revision process. The results suggest that using AWE as a supplement did not increase the amount of teacher feedback on content and that students appeared to value linguistic feedback from an instructor over a computer. Based on the CAF indices to examine writing quality, they found that learners could internalize the knowledge they accept from AWE to enhance their writing accuracy in both the short and long term. The online system, however, seems to fall short of teacher feedback when it comes to affecting writing complexity and fluency.

While the literature reviewed above is thought-provoking, the following aspects warrant further discussion. First of all, an overwhelming majority of studies have merely sampled learners at a single proficiency level (Ranalli, 2018). Moreover, how users respond to feedback across different categories when technology acts as scaffolding has remained under-explored. Additionally, non-corrective feedback, a salient property of *Pigai*, has rarely been noticed and discussed in prior literature. Finally, the study suggests CAF as the measurement to examine the impact of AWE on written production. In general, the present study aims to shed light on the intricate relationship between L2 proficiency, engagement with AWE feedback, and text quality. Specifically, the following research questions will be addressed:

1. How do students’ responses to different categories of AWE feedback vary across language levels?
2. How do the effects of AWE feedback on students’ writing quality vary across language levels?
3. How do students’ perceptions of AWE feedback vary across language levels?

## 3. Methods

### 3.1. Participants and contexts

A total of 42 first-year undergraduate students majoring in English from two intact classes at an inland Chinese university served as participants in this study. The first language of all participants is Chinese. Freshmen were chosen since they had no prior experience with AWE, and the two classes were considered because students were taught by the same teachers for all their specialized courses. It is worth noting that writing instruction was not covered in courses other than their writing course. The English writing course was scheduled for 1.5 h once a week and aimed to familiarize EFL learners with different genres of writing to improve their English writing skills including written expression, structuring sentences, paragraphs and essays, and developing content and ideas.

The language proficiency of the 42 participants was determined by two assessments. They were first categorized into two levels by their National College Entrance Examination (NCEE) English scores (150 points overall). Students who scored between 80 and 110 were placed at the low-intermediate level and those who scored between 110 and 140 were put at the high-intermediate level. The pre-test then reevaluated these two levels to further confirm the students’ writing proficiency. All participants’ pretest essay scores were based on the College English Test Band 4 (CET-4) writing criteria given by a professor with over 15 years of English teaching experience who has been involved in marking multiple CET-4 exams. The CET4 is a large-scale, high-stakes exam for Chinese university students and one that the participants in this study would soon be required to take, hence its scoring criteria are of high reliability and validity (Lei et al., 2023). The scores (*t* = −8.51, *p* < 0.001) eventually proved that Group A (*n* = 22) was low level and Group B (*n* = 20) was high level. After all participants signed the informed consent forms, their demographic information was collected including gender, age, years of English study and overseas learning experience. Specific background information for both groups is presented in Table 1. Because each writing task and pre- and post-test scores were included in 30% of their final course grade, no student failed to submit or complete their essays, so the final number of valid participants was 42.

**Table 1:** Demographic information for Group A and Group B.

		Group A (*n* = 22)	Group B (*n* = 20)
Gender	Female	20	18
	Male	2	2
Age		18.32	18.15
Years of English study		10.78	10.95
Overseas learning experience		None	None

### 3.2. The AWE system: *Pigai*

The AWE tool for students to submit drafts and receive guidance is called *Pigai*,^1^ which has been the largest online writing evaluation platform in mainland China. According to its webpage, *Pigai* has been used by more than 6,000 schools and universities, serving over 13 million students and reviewing over 220 million essays. After a user submits an essay, the system automatically assigns a score. The scoring model is calibrated from a large number of manually scored writings from a corpus (Bai and Hu, 2017). The score is generated by calculating the quantitative differences in vocabulary, sentence, structure and content between the user’s essay and the corpus texts, with the default percentages for the four dimensions being 43% for vocabulary, 28% for sentence, 22% for structure and 7% for content.

Apart from the holistic score, the system also provides an overall assessment and sentence-by-sentence reviews based on the algorithm (see Appendix A). The overall assessment covers four aspects of vocabulary, sentence, structure and content in the form of a comment. Sentence-by-sentence reviews identify grammatical errors that occur on a single-sentence basis and display them with red warning signs. In addition to corrective feedback, *Pigai* also gives non-corrective feedback, predominantly on synonyms, with the intention of embellishing language and acquiring language knowledge. The system searches the corpus based on a particular vocabulary in the user’s text to locate relevant words or phrases as supplementary material. This is a property of *Pigai*, yet few studies have measured its efficacy from the student’s perspective. Whenever a student revises content on the online platform and clicks submit, the platform re-evaluates the essay and records feedback points of each revision for the student and teacher to access. Students have access to the class ranking for each writing grade but are not authorized to view other students’ essays or scores. Teachers can manage their students’ texts only through their own registers to protect the privacy of their student’s data.

### 3.3. Data collection

Participants underwent a semester-long (14-week) experiment consisting of five AWE-intervention writing assignments and pre- and post-tests before and after the intervention. The procedure is shown in Table 2. All participants first completed a pre-test and then were trained in the operation of the *Pigai* software until proficiency was achieved. Afterwards, the course instructor assigned an essay that students were required to finish and submit to the online platform within 1 week and revise their drafts in the following week based on prompts from AWE, with no limit on the number of revisions. The process was run five times, and students only received tutorials from the AWE system. Finally, a post-test, questionnaires and interviews were immediately scheduled.

**Table 2:** Procedures of this study.

Week	Activities
2	Pre-test
3	Train
4	Submitting essay 1
5	Revising essay 1
…	
12	Submitting essay 5
13	Revising essay 5
14	Post-test
15	Questionnaire & Interviews

To ensure highly valid and reliable results, each task was subject to rigorous requirements in terms of word count, time limits, genre and topics. Writing must be completed within 40 min without the use of external resources. A minimum word count of 150 words was required. As the L2 writing instruction is primarily exam-oriented, it is acceptable for students to complete the required number of words in a limited amount of time. The genre for all writing was the argumentative essay, requiring the presentation of one’s arguments on a controversial social issue, which is the genre most practiced by Chinese English learners in high school and university classes. The essay topics were discussed by researchers and were all drawn from the CET-4 test pool with similar complexity. They are all familiar areas for students to guarantee fairness to each student.

After post-testing, all participants filled in a questionnaire with the aim of investigating their perceptions of the tool and collecting their learner profiles. The questionnaire was designed with 20 items (see Appendix B) in the form of a five-point Likert scale from 5 (strongly agree) to 1 (strongly disagree). These items were composed of four constructs: writing quality, cognitive engagement, behavioral engagement, and affective engagement. Items on writing quality were adapted from Wang et al. (2013), and items on cognitive engagement, behavioral engagement, and affective engagement were adapted from Han and Hyland (2015). Prior to the formal administration of the questionnaire, we performed a pilot test with five first-year university students majoring in English to ensure that all items were comprehensible, after which some items were slightly modified. In the main study, Cronbach’s alpha (α) for the four sub-scales were measured, namely writing quality (α = 0.91), cognitive engagement (α = 0.93), behavioral engagement (α = 0.88), and affective engagement (α = 0.91), indicating high reliability of the instrument (Cohen et al., 2007). Then, two subjects from each group were invited for stimulated recall interviews to explain the quantitative data in depth. By purposive sampling, we selected one student who frequently took up AWE feedback (Lily in Group A and Doris in Group B) and one who occasionally adopted AWE feedback (Joan in Group A and Chen in Group B) from each of the two groups to secure the gathering of abundant and representative information. Respondents’ AWE feedback and each writing version were readily presented to them to provoke reflection on their entire revision process. All interviewees were pseudonymous and had the right to withdraw from the study at any stage of data collection.

### 3.4. Data analysis

The data analysis process included textual analysis of student essays, AWE diagnostic information, questionnaires, and interview transcripts. Feedback points provided by *Pigai* to each participant and the acceptance of students in final versions were identified to answer the first question. According to the definition from Han and Hyland (2015), each written intervention that focuses on a different aspect is regarded as a feedback point, which was categorized into ten corrective feedback and three non-corrective feedback in this study. Among corrective feedback, mechanics refer to punctuation, capitalization, and spelling errors, and sentences include any inaccuracy in clause and sentence construction, such as run-ons, fragments, and word order (Ferris and Roberts, 2001). Non-corrective feedback attempts to tackle three dimensions: word, sentence, and organization. Instruction related to words is provided most frequently in synonyms, and feedback on sentences and organizations is presented in general comments. Due to the heavy workload, we selected three times (1st, 3rd, and 5th) of feedback from the first drafts and revisions from the final drafts for coding to obtain the feedback and uptake of the two groups at each feedback point to determine the discrepancies. It is important to note that the coding of uptake included students’ correct and incorrect revisions. To ascertain the reliability of data coding, approximately 20% of essays selected randomly were coded by two experienced and trained researchers to identify and categorize feedback provided by AWE and feedback adopted by students. The inter-coder agreement was 97% for feedback frequency and 85% for uptake frequency, and disagreements were resolved after discussion. Given that 80% agreement of coding is commonly recommended (Creswell, 2013), this study revealed good reliability of the data coding.

To answer the second question, in addition to the pre- and post-tests, the first drafts of the third writing task were included as a mid-test to examine the writing performance of high- and low-level students to strengthen the credibility of the results. Measuring dimensions include complexity, accuracy and fluency. Complexity is normally gauged in two subcategories: lexical complexity and syntactic complexity. They are two key measures of EFL learners’ written language output. In this study, lexical complexity and syntactic complexity are captured by the Lexical Complexity Analyzer (LCA) and L2 Syntactic Complexity Analyzer (L2SCA) developed by Lu’s team (Lu, 2011, 2012), which have been widely used in L2 writing research (e.g., Ai and Lu, 2013; Zhang and Cheng, 2021; Link et al., 2022; Fan, 2023). With the use of Python, these two computational tools automatically annotate texts in accordance with commands to compute complexity. According to Lu (2012), lexical complexity is composed of lexical density, lexical sophistication, and lexical diversity. Lu (2011) also conceptualized syntactic complexity as a multi-dimensional attribute of learners’ language use, including length of production, sentence complexity, subordination, coordination, and particular structures. Given the wide range of metrics and the possible duplication of information provided, the study selected a representative index from each category (see Table 3). Traditionally, the widely employed measure for assessing syntactic complexity and L2 writing is T-unit (Ellis and Yuan, 2004). To ensure the correct operation of computational tools, inter-sentence spaces, punctuation, and capitalization errors in students’ essays were manually amended to detect deep-seated linguistic features more precisely. Accuracy and fluency were obtained by manual labeling. We selected the EFC/C index proposed by Polio and Shea (2014) to evaluate accuracy by dividing the error-free clauses by the total number of clauses. Mechanics errors were excluded due to consideration of students’ rudimentary use of computers. The inter-coder reliability represented by the Intraclass Correlation Coefficient for accuracy was 0.89. Values between 0.75 and 0.90 are regarded as good reliability (Koo and Li, 2016). Fluency was measured by the total number of words produced in 40 min (Zhang and Cheng, 2021).

**Table 3:** CAF measures are used in this study.

Dimension	Measure	Label
Lexical complexity	Lexical density	LD
	Lexical sophistication	LS
	Lexical diversity	Uber
Global syntactic complexity	Mean length of T-unit	MLT
	Clauses per sentence	C/S
Specific syntactic complexity	Dependent clauses per T-unit	DC/T
	Coordinate phrases per T-unit	CP/T
	Complex nominals per T-unit	CN/T
Accuracy	Error-free clauses	EFC/C
Fluency	Total words	W

All text data were quantified for between-group comparisons. The data were detected for normal distribution, missing values, outliers, and equality of variances preceding the statistical analysis. No missing values and outliers were found, and all variables conformed to normality. According to Field (2013), the z-scores for skewness and kurtosis did not exceed 1.96, and the data were presumed to be normally distributed. Levene’s Test was taken to check the assumption of equality of variances. The results showed that all indices satisfied the assumption except for DC/T in the pre-test, DC/T and CP/T in the mid-test, and Writing Quality. In independent samples t-tests, variables for which the Null hypothesis of equal variances is rejected can be read out by the Welch procedure (equal variances not assumed) (Larson-Hall, 2016). In this study, independent samples t-tests were performed on the pre-, mid-, and post-test data to examine the differences in the transfer effects of AWE on each index between the two levels. For effect sizes, Cohen (1988) states that *d* threshold values of 0.2, 0.5 and 0.8 correspond to small, medium and large effects, respectively. Data from the questionnaire and interviews were analyzed in response to the third question. The study used independent samples t-tests to identify whether the two groups perceived AWE differently from the four constructs. Interviews served as supporting evidence for the questionnaire to be explored further.

## 4. Major findings

### 4.1. Comparison of revision responses to AWE feedback

Tables 4, 5 were drawn to better illustrate the properties of *Pigai* and the responses of students with different L2 proficiency to its feedback. The two tables reflected the frequencies of feedback identified by *Pigai* based on the first drafts and the frequencies of uptake in the final drafts by low- and high-level students, respectively. It is evident from Table 4 that the errors detected by the system in Group A gradually decreased over time. Although adoption varied from time to time, the uptake rates of corrective feedback were globally high, remaining around 70%. Specifically, participants in Group A were more likely to make mechanics-related errors all three times, followed by sentence and collocation-related errors. The system provided significantly more non-corrective feedback with a recommendatory nature than corrective feedback for error recognition, and the reduction in total feedback was also negligible. However, these recommendations did not attract as much attention from students as the false warnings, which were accepted by approximately 10% each time. About all of the output was at the lexical level, whereas the sentence and paragraph levels received very few comments.

**Table 4:** Feedback and uptake frequencies for three tasks in Group A.

	Time 1		Time 3		Time 5	
Feedback point	Feedback frequencies	Uptake frequencies	Feedback frequencies	Uptake frequencies	Feedback frequencies	Uptake frequencies
Mechanics	75	65	49	37	27	24
Article	11	6	8	6	5	3
Preposition	7	5	4	3	5	3
Pronoun	1	1	2	1	0	0
Noun	15	9	7	4	7	4
Verb	11	7	15	6	8	5
Adjective	2	2	0	0	0	0
Adverb	1	1	0	0	1	1
Collocation	15	7	19	10	11	6
Sentence	31	22	23	16	13	8
Total	169	125 (73.97%)	127	83 (65.35%)	77	54 (70.13%)
Word	391	52	363	36	373	43
Sentence	15	1	10	3	12	3
Paragraph	4	0	1	1	3	1
Total	410	53 (12.93%)	374	40 (10.70%)	388	47 (12.11%)

**Table 5:** Feedback and uptake frequencies for three tasks in Group B.

	Time 1		Time 3		Time 5	
Feedback Point	Feedback frequencies	Uptake frequencies	Feedback frequencies	Uptake frequencies	Feedback frequencies	Uptake frequencies
Mechanics	49	40	29	20	18	13
Article	9	7	7	4	3	3
Preposition	3	2	1	1	5	3
Pronoun	2	2	1	0	1	0
Noun	7	3	4	4	3	2
Verb	12	8	13	7	5	3
Adjective	0	0	0	0	0	0
Adverb	0	0	0	0	1	0
Collocation	10	6	11	7	13	7
Sentence	19	16	14	11	10	7
Total	111	84 (75.68%)	80	54 (67.50%)	59	38 (64.41%)
Word	380	77	353	63	351	87
Sentence	10	3	7	3	6	4
Paragraph	2	2	3	1	1	1
Total	392	82 (20.92%)	363	67 (18.46%)	358	92 (25.70%)

As shown in Table 5, Group B experienced a similar trend in that the errors identified by the tool were reduced slightly. Nevertheless, the difference was that the total amount of corrective feedback was significantly less in all three cases than in Group A. Group B showed a decreasing trend in uptake with 75.68, 67.50 and 64.41%, respectively. Participants presented a picture of a high amount of non-corrective feedback but a low amount of adoption; however, the adoption rate was also higher than that of Group A, reaching around 20%.

### 4.2. Comparison of writing quality affected by AWE feedback

Tables 6, 7 record descriptive statistics including means and standard deviations of the ten CAF measures in the three tests for lower- and upper-level students, respectively. To compare the writing performance of the two groups at each phase of the AWE intervention, independent samples t-tests were executed. Table 8 contains *t*-values, *p*-values, and Cohen’s d values for each index in the pre-test, mid-test, and post-test. Before the AWE intervention, pretest results demonstrated that high-level students (Group B) outperformed low-level learners (Group A) in lexical density LD (*p* < 0.01, *Cohen’s d* = −1.18) and accuracy EFC/C (*p* < 0.05, *Cohen’s d* = −0.71). However, no significant between-subject differences were found for other measures (LS: *p* = 0.717; Uber: *p* = 0.347; MLT: *p* = 0.674; C/S: *p* = 0.191; DC/T: *p* = 0.138; CP/T: *p* = 0.618; CN/T: *p* = 0.798; W: *p* = 0.613).

**Table 6:** Descriptive statistics of CAF measures in Group A.

	Pre-test		Mid-test		Post-test	
	*M*	SD	*M*	SD	*M*	SD
*Lexical Complexity*						
LD	0.47	0.03	0.48	0.04	0.50	0.04
LS	0.47	0.08	0.45	0.10	0.46	0.05
Uber	20.43	3.17	22.44	2.73	21.82	2.87
*Syntactic Complexity*						
MLT	14.43	2.06	15.09	1.50	14.68	1.74
C/S	1.43	0.21	1.46	0.18	1.53	0.22
DC/T	0.32	0.14	0.39	0.18	0.42	0.19
CP/T	0.62	0.23	0.51	0.33	0.43	0.20
CN/T	1.50	0.34	1.56	0.29	1.53	0.30
*Accuracy*						
EFC/C	0.39	0.14	0.49	0.15	0.51	0.15
*Fluency*						
W	171.36	21.72	178.32	23.12	174.73	22.93

**Table 7:** Descriptive statistics of CAF measures in Group B.

	Pre-test		Mid-test		Post-test	
	*M*	SD	*M*	SD	*M*	SD
*Lexical Complexity*						
LD	0.50	0.02	0.49	0.05	0.51	0.03
LS	0.48	0.06	0.47	0.09	0.51	0.08
Uber	21.31	2.76	24.25	2.63	23.91	2.69
*Syntactic Complexity*						
MLT	14.69	1.87	15.39	1.60	14.96	2.14
C/S	1.50	0.14	1.62	0.25	1.60	0.28
DC/T	0.41	0.23	0.50	0.27	0.57	0.25
CP/T	0.66	0.26	0.40	0.18	0.49	0.19
CN/T	1.47	0.38	1.61	0.29	1.57	0.31
*Accuracy*						
EFC/C	0.50	0.17	0.56	0.15	0.54	0.17
*Fluency*						
W	168.30	16.53	182.85	21.57	186.75	29.61

**Table 8:** Independent samples *t*-tests for CAF between Group A and Group B.

	Pre-test			Mid-test			Post-test		
	*t*	*p*	*Cohen’s d*	*t*	*p*	*Cohen’s d*	*t*	*p*	*Cohen’s d*
*Lexical Complexity*									
LD	−3.57	0.001^**^	−1.18	−0.22	0.825	−0.22	−1.84	0.073	−0.28
LS	−0.37	0.717	−0.14	−0.64	0.525	−0.21	−2.33	0.025*	−0.75
Uber	−0.95	0.347	−0.30	−2.19	0.035^*^	−0.68	−2.43	0.020*	−0.75
*Syntactic Complexity*									
MLT	−0.42	0.674	−0.13	−0.64	0.528	−0.19	−0.46	0.650	−0.14
C/S	−1.33	0.191	−0.39	−2.38	0.022^*^	−0.74	−0.96	0.343	−0.28
DC/T	−1.52	0.138	−0.47	−1.57	0.125	−0.48	−2.18	0.035^*^	−0.68
CP/T	−0.50	0.618	−0.16	1.31	0.199	0.41	−0.94	0.351	−0.31
CN/T	0.26	0.798	0.08	−0.57	0.574	−0.17	−0.46	0.652	−0.13
*Accuracy*									
EFC/C	−2.28	0.028*	−0.71	−1.36	0.180	−0.47	−0.61	0.546	−0.19
*Fluency*									
W	0.51	0.613	0.16	−0.66	0.516	−0.20	−1.48	0.147	−0.45

^**^p < 0.01; ^*^p < 0.05.

After a duration of the AWE intervention, the two groups’ performance in terms of lexical density and accuracy converged gradually. In the mid-test, no significant differences were observed for most metrics (LD: *p* = 0.825; LS: *p* = 0.525; MLT: *p* = 0.528; DC/T: *p* = 0.125; CP/T: *p* = 0.199; CN/T: *p* = 0.574; EFC/C: *p* = 0.180; W: *p* = 0.516), but Group B was higher than Group A on lexical diversity Uber (*p* < 0.05, *Cohen’s d* = −0.68) and sentence sophistication C/S (*p* < 0.05, *Cohen’s d* = −0.74).

In the posttest, there were no differences between the two groups in accuracy (*p* = 0.546) and fluency (*p* = 0.147). However, Group B differed much from Group A on lexical dimensions, as illustrated by lexical sophistication LS (*p* < 0.05, *Cohen’s d* = −0.75) and lexical diversity Uber (*p* < 0.05, *Cohen’s d* = −0.75). At the syntactic level, no statistically significant differences were identified between the two groups, except for the measure related to subordinate clauses, DC/T (*p* < 0.05, *Cohen’s d* = −0.68).

### 4.3. Comparison of perceptions toward AWE feedback

Two sets of data collected from the four constructs of the questionnaire were treated with descriptive statistics and independent samples t-tests to explore how different levels of students perceive this type of technology-assisted feedback (see Table 9). These two groups of participants showed significant differences in all four dimensions. Compared to advanced students, less advanced students held a more favorable belief about the impact of AWE feedback on improving writing quality (*p* < 0.001, *Cohen’s d* = 1.46). In relation to cognitive engagement, it appears that skilled students exhibited higher levels of cognitive and metacognitive strategies when interacting with AWE compared to unskilled students (*p* < 0.05, *Cohen’s d* = −0.70). Likewise, the two groups showed significant differences in behavioral engagement, reflected in the fact that high-level students perceived performing more revision operations when confronted with AWE feedback (*p* < 0.05, *Cohen’s d* = −0.73). However, lower-level learners significantly had a more positive attitude and emotion toward AWE than higher-level students (*p* < 0.01, *Cohen’s d* = 0.91).

**Table 9:** Independent samples *t*-tests for perceptions between Group A and Group B.

	Group A (*n* = 22)	Group B (*n* = 20)	*t*	*p*	*Cohen’s d*
	M ± SD	M ± SD			
Writing Quality	3.74 ± 0.61	2.41 ± 1.14	4.428	0.000^***^	1.46
Cognitive Engagement	2.79 ± 1.19	3.59 ± 1.10	−2.253	0.030^*^	−0.70
Behavioral Engagement	2.72 ± 1.00	3.53 ± 1.20	−2.399	0.021^*^	−0.73
Affective Engagement	3.72 ± 1.01	2.74 ± 1.14	2.956	0.005^**^	0.91

^***^p < 0.001; ^**^p < 0.01; ^*^p < 0.05.

Interview manuscripts were provided as supporting evidence of the quantitative data to explain the motives behind it. In Group A, Lily believed this AWE system was a good writing assistance tool that was applied by her as grammar correction software. She modified all the feedback with warning signs, yet the learning resource feedback was hardly responded to. She emphasized the significance of writing accuracy to her at the current stage, and the AWE feedback enabled her to be aware of the common errors she made to mitigate future risks. As she described:

*The feedback informed me that the most frequent error was the singular-plural form, which made me more alert to the correct use of nouns in my future writing and to develop an awareness of proofreading after writing.*

Joan claimed that she preferred the more explicit information as she could quickly capture errors and correct them. But her current lack of language proficiency probably led to her deficient comprehension of implicit suggestions from the system, particularly certain intractable language points, like sentence structure. Consequently, she eventually decided to forgo the intervention. She described the AWE feedback in a few words: vague, confusing, and generic.

It is interesting to find that both high-level students interviewed were suspicious of the potential of AWE feedback to improve writing performance and language development. Chen indicated that he attached great importance to both corrective and non-corrective feedback, treating them as a vital channel in the acquisition of English. However, he expressed his distrust of the precision of feedback and did not blindly adopt all suggestions. Conversely, he only revised what he considered correct and appropriate. As he conveyed in the interview:

*I have to admit that the feedback given is not entirely accurate. For example, my sentence It is requisite for children to exchange ideas with parents from time to time was assessed as having the issue of fragmentation. However, I am sure that the sentence pattern I used is accurate. Thus I chose not to make any changes.*

Besides correcting errors, students also polished the language according to non-corrective feedback, focusing on synonym expressions. Chen stated that he pondered whether the recommended synonym fit the sentence context or was simply a low-frequency word. After accepting appropriate expressions, the rest of the learning resources were read as extended knowledge. We noticed a similar pattern in Doris’s revision process, in which errors were corrected first, and then the language was improved. One exception was that she was also concerned with the meaning-level linguistic features. Accordingly, she has taken general comments into account in her essay revision. As she indicated:

*The system advised me to increase the use of complex sentences and transitional words. I knew the weight of sentences and cohesion on the quality of a text. Therefore, with the help of dictionaries and online resources, I managed to add some appropriate subordinate clauses and logical connectors to my text.*

Unfortunately, Doris expressed a lack of AWE feedback on the meaning level and her desire for more precise instruction to improve her language rather than an emphasis on language accuracy.

## 5. Discussion

This study is motivated by the need to better understand the complex interactions between computerized feedback, language proficiency, revision processes, and writing products. To accomplish this goal, we seek to observe what linguistic feedback *Pigai* gives and how students with varying L2 levels react to it. We attempt to discover how learners at different levels perceive it, what factors influence their decisions, as well as whether and how feedback plays a role in writing quality. The diagnosed corrective feedback showed a declining tendency as the number of interventions grew, and this trend was followed by a reduction and a rebound in uptake rates from high-level and low-level learners. Unexpectedly, low-level students appear to pay more attention to revising errors than high-level users. In conjunction with the questionnaire and interview results, this discrepancy in reactions may be explained by the idea that lower-level students were driven by the advancements that come with treatable feedback, while higher-level students were demotivated by more stable performance and restricted accuracy growth, supporting Xu and Zhang’s (2022) view. Additionally, the precision of AWE feedback should be considered as a hindrance for high-level students to make further responses. Overall, the adoption of corrective feedback is high at both levels, as asserted in Lavolette (2014). Among them, type-related revisions are the most frequent and effective, which underpins the finding of Stevenson et al. (2006). The inconsistent responses to mechanical and grammatical feedback points somewhat validate Storch and Wigglesworth’s (2010) argument that more superficial errors (e.g., errors in mechanics) cost learners less effort, while grammatical errors require higher and long-term engagement to be understood and internalized.

Given the nature of the system, it can automatically provide a considerable amount of non-corrective feedback from the corpus based on the recognition of a word or phrase in students’ texts. Nevertheless, data collected from students’ three written tasks exposed a low take-up rate. Feedback on vocabulary accounted for almost all, while fewer concerns were given to sentences and organization. As a result, students are restrained to lexical-level revisions when faced with non-corrective feedback. It is evident that higher achievers engage more with non-corrective feedback than lower achievers, manifesting in deeper linguistic awareness to develop writing. In general, error correction remains a top priority in Chinese EFL students’ perceptions of writing development, but high-proficiency students have developed a relatively sophisticated awareness of lexical and morphosyntactic aspects.

Differences in CAF between students with different proficiency levels on the pretest, midtest, and posttest were examined to elucidate the effect of computerized feedback on written quality while mediated by language level. After one semester of utilizing the system, the higher-level students’ advantage in accuracy and lexical density vanished, but lexical sophistication and diversity improved. The refinement in the accuracy of low-level students demonstrates the efficacy of AWE feedback in language error reduction (Wang et al., 2013; Xu and Zhang, 2022), but the change from significant disparity to no disparity between different L2 levels points out its greater effect on the writing accuracy of unskilled learners. According to Maamuujav (2021), learners with proficiency gaps generally exhibit differences in lexical density. Less skilled students’ increased lexical density may be a sign that AWE feedback can facilitate learners to enrich the information carried by sentences. The lexical variation reveals that the long-term uptake of lexical-related feedback, especially synonyms and collocations, could augment text richness. Students with better competence, in contrast to students with lesser proficiency, have established an awareness of choosing appropriate content words and have tended to pursue advanced words suggested by the system as alternatives to commonly used ones, resulting in enhanced lexical rareness. Consequently, there is an interaction between the feedback from *Pigai* and learners’ language level to promote the use of word chunks and to improve the lexical complexity of essays. However, the impact may vary depending on the language proficiency of students, and more advanced students are likely to benefit more.

Apart from that, the syntactic level also produced changes, which were reflected in the differences in the C/S and DC/T measures between skilled students and unskilled students. These two indices imply that advanced writers have increased the use of subordinate clauses in their writing, which is a sign of language development. As learning time and language level grow, the syntactic complexity of L2 learners generally follows a trajectory from reliance on coordinated structures to subordinate clauses and to extended nominal forms (Ai and Lu, 2013; Biber et al., 2016; Yoon and Polio, 2017). It may be inferred from the variation in clausal use that high-level learners who receive AWE feedback are more likely to elicit metacognitive and cognitive operations in the processing stage, which aligns with Zhang (2020). Given the observations of revised versions, advanced students tended to adopt more complex structures to achieve sentence reconstruction when faced with prompts, such as missing conjunctions and run-on, rather than the simple substitution of conjunctions or commas. It is noteworthy that only some micro-metrics showed changes, but no discrepancies were found in the MLT and C/S indices in the mid- and post-tests, which represent holistic syntactic complexity. Finally, there was no significant difference in fluency between the two levels of learners. Fluency is a measure of a learner’s ability to automate their L2 linguistic knowledge in real times (Wolfe-Quintero et al., 1998). It might evolve slowly since it is more dependent on the psycholinguistic process when applying L2 knowledge (Housen and Kuiken, 2009).

Analysis of the questionnaire and interview transcripts revealed that students with different proficiency levels appeared to have varying perceptions and beliefs when coping with AWE-supported feedback. It is surprising to find that low-level students recognize the contribution of automated feedback to the improvement of writing quality more than high-level students. Advanced students complained about the precision of feedback and the lack of feedback on meaning-level language features (e.g., syntax, structure, content development) that did not satisfy them for long-term language enhancement at a higher level. According to Stevenson et al. (2006), resource squeezes that originate from low-level revisions may affect resources available for other processing. From the perspective of engagement, proficient students generally present higher cognitive and behavioral engagement compared to less proficient learners. They tend to understand AWE feedback rather than merely notice it as well as implement a range of revision strategies, thus automated feedback could help develop their writing in some way. The finding further supports the conclusions of the first and second questions as well. In contrast to the less positive perceptions about the AWE system from high-level students, low-level students express a more supportive attitude, paying excessive attention to correct errors and improve accuracy. Overall, feedback given by AWE serves as a new kind of mediation that scaffolds learners’ revision and writing process (Jiang et al., 2020). But it should be noted that only when learners effectively engage in AWE feedback can they really benefit from it (Zhang and Hyland, 2022). The interviews made it clear that individual or contextual factors such as learning beliefs, revision motivation, learning strategies, and metacognitive competencies of students with different proficiency levels could affect the extent of their engagement and productivity.

## 6. Conclusion

This study enriches the line of research on AWE and CALL in L2 writing. The results reveal some similarities and differences between lower- and higher-level students when encountering automated feedback and the strengths and weaknesses of AWE systems. There are several implications for *Pigai* and other similar AWE tools and for the future pedagogy of L2 writing. First, AWE system developers should focus more on how to raise the precision of corrective feedback and the recognition of syntactic complexity and higher-level language features to upgrade the current program. Corrective feedback has been the first intention of students using AWE tools to tutor their writing, hence improving the pertinence and accuracy of the feedback is essential. Beyond this, error correction may not keep English learners relying on AWE systems for long, thus the provision of higher-level writing feedback is more likely to enhance the efficacy of AWE for student writing improvement. As such, system developers must be aware of the current issues with CALL. Promoting the upgrade of AI technology enables English teaching and learning more efficient and accessible and realizes education empowered by AI. The study is also instructive for teachers in the L2 instruction process, advising them on how to apply AI technology properly and sensitizing them to the contributions of AWE to students’ writing skills and language acquisition. Teachers are recommended to integrate AWE feedback into the L2 writing teaching based on a thorough understanding of its merits and demerits (Jiang et al., 2020). This study indicates the mediating effect of language proficiency in revision processes and writing outcomes, which should also be taken into account. AWE feedback that visually shows students’ frequent errors can assist teachers in increasing relative grammar instruction and practice in writing teachings, such as sentence structures, collocations, and verb errors found in this study. Given the predominantly error-correcting nature of automated feedback and the emphasis students place on surface revision, it is necessary for teachers to teach complex subordinate clauses, coherence, contents, and other components of writing that are marginalized by AWE systems to transform students’ perceptions and promote their overall development of writing competence. In other words, teachers should adapt their teaching strategies to match their students’ learning beliefs (Huang et al., 2020). Teachers are expected to realize that AWE feedback serves as a helpful supplement rather than a replacement of other feedback sources (Bai and Hu, 2017; Link et al., 2022), which should be flexibly combined with the teacher and peer feedback to maximize its advantages. Educators should bear in mind their guiding role as well as the function of technology in assisting them, which means the value of a win-win, coordinated development of technology and education.

Understandably, this study has its own limitations. Although this study has observed the dynamic development of CAF linguistic features among EFL learners of different proficiency levels, the design lacked a control group, weakening the reliability of the causality between writing improvement and the use of automated feedback. A control group without any intervention will be included in future studies as a comparison. Additionally, the study suffered from a relatively small number of participants. For instance, the inadequacy of the sample size in both the pilot and formal testing phases of the questionnaire could have restricted the ability to extrapolate perceptions regarding computer-generated feedback to a broader audience. In future research, efforts will be made to bolster sample sizes, and more advanced tools such as think-aloud protocol and eye-tracking software might be adopted to capture more student engagement and its relationship to writing performance.

## Data availability statement

The original contributions presented in the study are included in the article/supplementary material, further inquiries can be directed to the corresponding author.

## Ethics statement

The studies involving humans were approved by Xi’an Jiaotong University City College. The studies were conducted in accordance with the local legislation and institutional requirements. The participants provided their written informed consent to participate in this study.

## Author contributions

CS: Conceptualization, Formal analysis, Methodology, Writing – original draft, Writing – review & editing. PS: Formal analysis, Methodology, Writing – review & editing. JG: Formal Analysis, Funding acquisition, Writing – review & editing. SX: Conceptualization, Methodology, Writing – review & editing. JT: Methodology, Writing – review & editing.

## Figures and Tables

**Figure A1:**
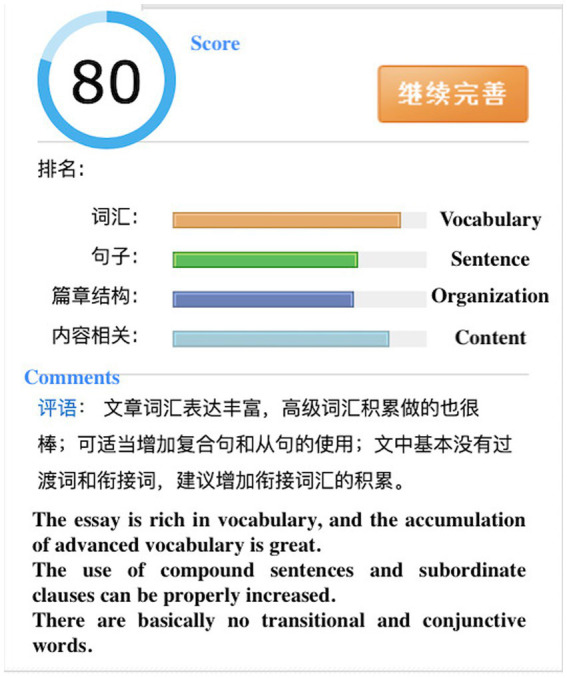
A screenshot of the score and general comment in Pigai.

## Footnotes

^1^http://www.pigai.org/

## Appendix A

Figure A1

## Appendix B

Questions from the questionnaire:

*The dimension of writing quality*

AWE feedback helps me correct grammatical errors in essays.

With the help of AWE feedback, I try to use different words in essays.

With the help of AWE feedback, I try to use different sentence structures in essays.

With the help of AWE feedback, I write essays faster.

AWE feedback helps me improve my writing quality.

*The dimension of cognitive engagement*

It is easy for me to understand the feedback provided by the system.

Based on system feedback, I know how to revise essays to improve the writing quality.

AWE feedback helps me acquire grammatical knowledge that I can use in future writing.

I often incorporate other learning resources or tools to revise essays.

AWE feedback helps me realize the strengths and weaknesses of my current writing.

*The dimension of behavioral engagement*

I usually spend a lot of time analyzing feedback and revising my essays.

I read evaluation reports carefully and make a lot of changes based on the feedback.

Based on feedback, I usually make a lot of changes in grammar.

Based on feedback, I usually make a lot of changes in vocabulary and sentence patterns.

Based on feedback, I usually make a lot of changes in structure and content.

*The dimension of affective engagement*

I enjoy the process of revising essays based on system feedback.

I feel a sense of pride and satisfaction after revising essays based on feedback.

AWE feedback helps increase my interest and motivation in writing.

AWE feedback helps improve my overall writing skills.

I would like to continue using AWE in the future.
